# Statistical prediction of interactions between low concentrations of inhibitors on yeast cells responses added to the SD-medium at low pH values

**DOI:** 10.1186/s13068-019-1453-4

**Published:** 2019-05-09

**Authors:** Cecilia Laluce, Longinus I. Igbojionu, José L. Silva, Clóvis A. Ribeiro

**Affiliations:** 1 Institute of Research in Bioenergy (IPBEN), Institute of Chemistry, São Paulo State University (UNESP), R. Prof. Francisco Degni, 55, Araraquara, SP CEP 14800-060 Brazil; 2 Dept Analytical Chemistry, Institute of Chemistry, State University of São Paulo, Júlio de Mesquita Filho-UNESP, R. Professor Francisco Degni, 55, Araraquara, São Paulo CEP 14800-060 Brazil

**Keywords:** *Saccharomyces cerevisiae*, Biomass inhibitors, Factorial designs, RMS analysis, Response optimizer, Interactions between inhibitors

## Abstract

**Background:**

In the present work, the main inhibitors of the yeast cells (vanillin, furfural, formic, and levulinic acid) were generated by pretreatments or hydrolysis (sulfuric acid or enzymes) to convert reducing sugars into ethanol. Inhibitors were added at increasing concentrations to the SD-medium containing yeast extract while negative effects on yeast cells were observed. Statistical analyses were applied to predict and interpret results related to biomass production.

**Results:**

Inhibitors affected productivities and yields of biomass and ethanol when added to SD-medium. Based on the 2^3^ full-central-composite design, “predicted” and “observed” values of ethanol and biomass were obtained in presence of the major inhibitors, which were acetic acid, formic acid, and levulinic acids. Increases in biomass and ethanol production are described in the Response surface graphs (RSM graphs) that resulted from multiple interactions between inhibitors. Positive interactions between the inhibitors occurred at low concentrations and pH values. The results were experimentally validated.

**Conclusions:**

Statistical analysis is an extremely useful tool for predicting data during process monitoring, while re-adjustments of conditions can be performed, whenever necessary. In addition, the development of new strains of yeast with high tolerance to biomass inhibitors will have a major impact on the production of second-generation ethanol. Increases in fermentation activity of the yeast *Saccharomyces cerevisiae* in a mixture containing low concentrations of inhibitors were observed.

## Background

The conversion of agricultural wastes into ethanol is a rapidly developing technology. Biomasses are cheap, abundant, and unique materials for the production of bioenergy of low cost at industrial scale [1].

Typically, most of the lignocellulose biomass is comprised of about 10–25% lignin, 20–30% hemicellulose, 40–50% cellulose, and 2.4% ash [2–6]. In addition, lignocellulose biomass is one of the main agricultural crops of tropical countries, with world production of about 1.6 billion tons that corresponds to about 279 million tons of residues, including bagasse and leaves [7, 8]. High productivity and low cost efficiency are the characteristics of an economical and efficient process of utilizing vegetal biomass [9].

Several key factors contribute to increase the recalcitrance of biomass materials and they are the following [10, 11]:

(i) high lignin content; (ii) high contents of hemicellulose; (iii) protection of the cellulose component by lignin; (iv) strong fiber strength; (v) difficult access of chemicals and enzymes to surface areas. In addition, the composition of cellulose, hemicellulose, and lignin varies from one plant species to another [12].

Agricultural residues are mainly composed of cellulose, hemicellulose, and lignin, along with small amounts of other components, such as acetyl groups, minerals, and phenolic constituents present in lignin [13].

### Pretreatments of biomasses

An appropriate pre-treatment method applied prior to the enzymatic saccharification of the cellulose improves the efficiency of the whole processes [14]. Pretreatments usually degrade the hemicellulose leading to the formation of by-products from pentose and hexose, such as sugar acids, aliphatic acids (mainly acetic acid, formic acid, levulinic acid), aliphatic carboxylic acids, and furan aldehydes such as hydroxy-methyfurfural (HMF) [15, 16]. In minimal medium, growth rates and ethanol production are generally low at pH 4.5 in the presence of acetic or lactic acid within the range of 0.05–0.1% (w/v), as describe in literature [17].

Depending on conditions, the fermentation of the glucose by the yeast *Saccharomyces cerevisiae* in presence of biomass inhibitors can vary as follows [18]: vanillin > phenol > 5-HMF > furfural > levulinic acid > acetic acid > acid formic. The strongest inhibition of the conversion of biomass into ethanol is usually vanillin.

### Process responses and statistical methods

The response variables of ethanol production such as yields of ethanol and biomass formation and viability are dependent on the Statistical Planning adopted. Independent variables are the concentrations of inhibitors (acetic acid, formic acid, levulinic acid, furfural, and vanillin acids).

The selection of a minimum number of experiments is an essential factor for the good progress of the research [19]. Statistical interpretation of the experimental results is inherent in the research process and the control of the experimental variables can reduce the experimental error. The knowledge of statistical tests and assumptions are equally critical to make the research statistically valid [19].

The objective of the present work was to determine the eventual positive and/or negative interactions between fermentation responses (yeast growth, ethanol production, and viability) in the presence of the low amounts biomass inhibitors using statistical methods. High inhibitory effect was also observed in the presence of vanillin during the conversion of biomass residues into the second-generation ethanol [18].

## Results and discussion

### Effects of increasing concentrations of inhibitors added to the SD-medium on main responses of yeast cells

**Fig. 1:**
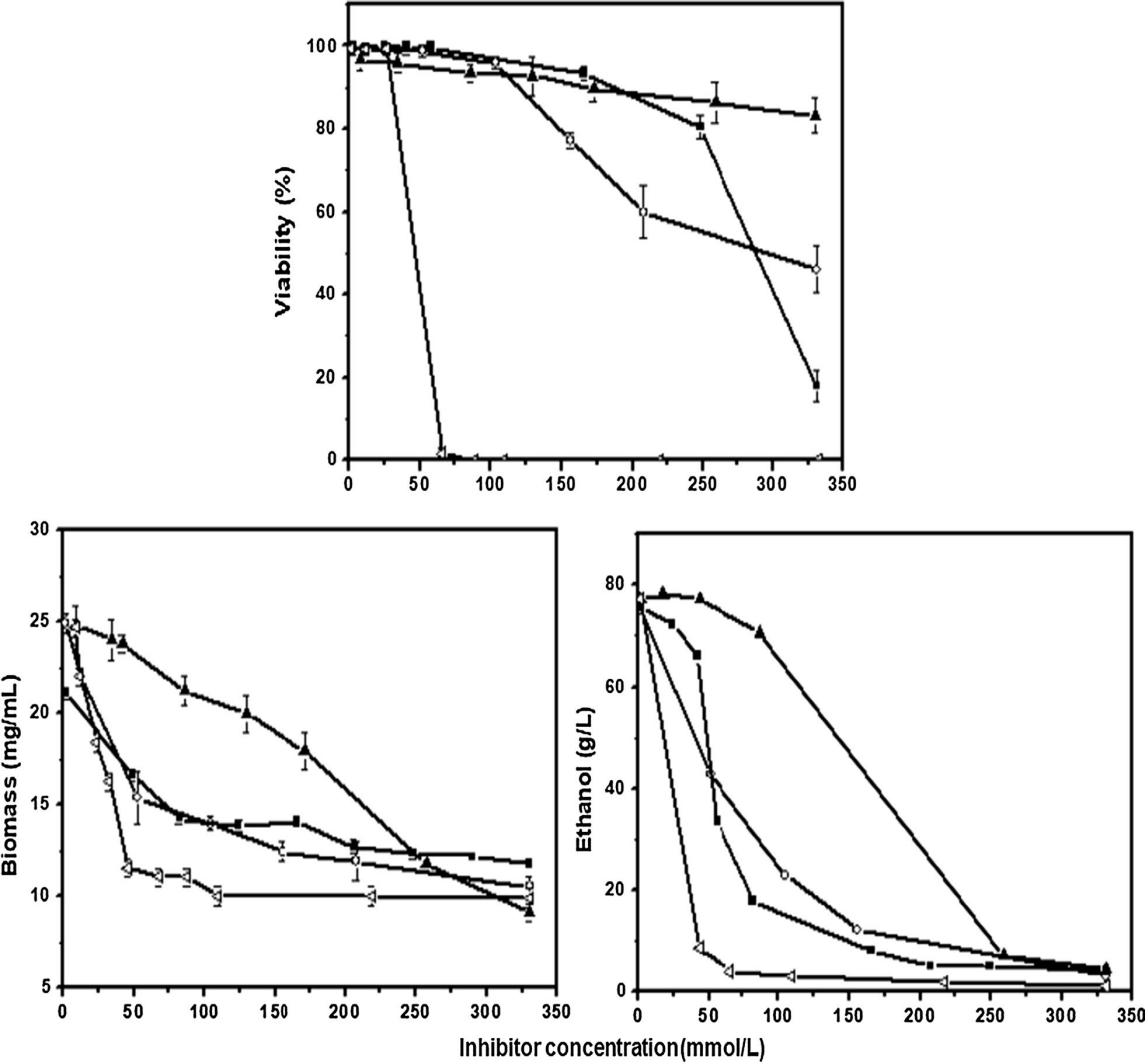
Yeast responses to the biomass inhibitor added to SD-medium were biomass (mg/mL), ethanol (g/L), and viability (%) formic acid, and levulinic acid (black up-pointing triangle), while the symbols used to represented the inhibitor were: formic acid (white left-pointing triangle), acetic acid (white circle), furfural (black square), and levulinic acid (black up-pointing triangle)

In Fig. 1, fermentation responses (biomass, ethanol, and viability) were determined for initial concentrations of inhibitors added to the medium ranging from zero to 350 mmol/L. Dramatic reductions in biomass, ethanol and viability were observed in presence of the four inhibitors at concentrations above 50 mmol/L. On the other hand, higher inhibitory effects on formation of biomass (mg/mL), ethanol (g/L), and viability (%) were obtained in presence of formic acid.

In the presence of levulinic acid varying from 100 to 98% the lowest inhibitory effects on viability were obtained, while decreases in viability were observed in the presence of furfural, formic acid, and acetic acid.

### The effects of increasing concentrations of inhibitors added to SD-medium on yields and productivities of biomass and ethanol

The effects of increasing the concentrations of the four inhibitors (acetic, formic, furfural, and levulinic acids) on the yields of ethanol (*P*, g/L) and biomass (*X*, mg/L) are described in Table 1. When concentrations of inhibitors increased, there was a dramatic decrease in ethanol formation than on biomass. The obtaining high levels of energy required for growth and other cellular activities are the main goal of fermentation. Slow fermentations may be a risk to the viability of the yeast cells on an industrial scale due to significant decreases in the specific growth rate of the yeast cells at low pH values [20]. The use of fast, cheap, and efficient methodologies to disintegrate and hydrolyze the lignocellulosic biomass is the major challenge of the production of the second-generation ethanol [21].

**Table 1:** Effects of initial concentrations of inhibitors on the ethanol (*Q*_P_) and biomass (*Q*_X_) productivities, ethanol (*P*), and biomass (*X*) yields

Inhibitors		Ethanol		Biomass	
Inhibitors	(mm/L)	*P* (g/L)	*Q*_P_ (g/L/h)	*X* (mg/L)	*Q*_X_ (g/L/h)
w/o inhibitor	–	75.0 ± 0.3	12.5 ± 0.8	24.7 ± 0.8	4.1 ± 0.9
Acetic ac.	24.8	72.2 ± 1.4	12.0 ± 0.1	20.6 ± 0.1	3.4 ± 0.1
	82.6	18.0 ± 1.3	3.0 ± 0.4	14.0 ± 0.1	2.3 ± 0.2
	165.3	8.3 ± 0.1	1.4 ± 0.1	13.6 ± 0.5	2.3 ± 0.1
Formic ac.	21.7	78.3 ± 2.8	11.9 ± 0.6	18.5 ± 0.2	3.1 ± 0.2
	86.9	4.1 ± 0.2	0.7 ± 0.1	10.5 ± 0.1	1.8 ± 0.1
	108.6	3.0 ± 0.1	0.5 ± 0.1	9.9 ± 0.4	1.7 ± 0.1
Furfural	52.2	42.1 ± 0.5	7.0 ± 0.1	15.3 ± 1.5	2.6 ± 0.2
	104.1	22.8 ± 0.8	3.8 ± 0.1	13.9 ± 0.1	2.3 ± 0.1
	208.1	4.8 ± 0.4	0.8 ± 0.1	11.8 ± 1.1	2.0 ± 0.1
Levullinic ac.	86.1	71.0 ± 0.2	11.8 ± 0.4	21.5 ± 0.7	3.6 ± 0.1
	129.2	54.6 ± 0.4	9.1 ± 0.1	19.6 ± 1.0	3.3 ± 0.2

Fermentations were performed at initial cell density of 10.0 mg/mL in the SD-medium at pH initially adjusted to 4.5. Means (± SD) of data resulted from experimental repetitions

### Pareto analysis

**Fig. 2:**
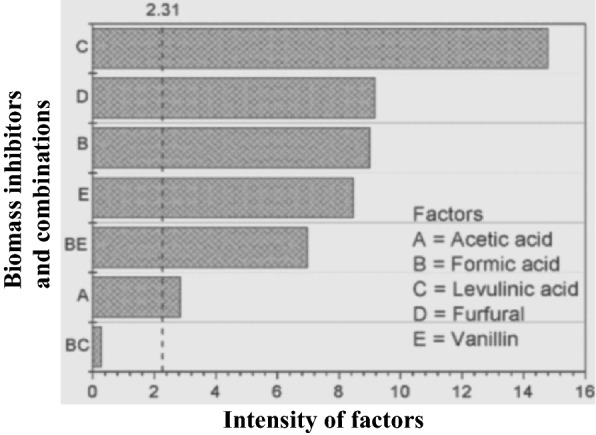
Pareto chart (absolute values represented in the *Y*-axis) and the vertical line (reference line) that indicates effects of the biomass inhibitors above reference line

As shown in Fig. 2, Pareto analysis was performed to compare the effects of the inhibitors (*Y*-axis) with the intensities of inhibitions (*X*-axis). The independent variables plotted on the ordinate (*Y*-axis) were *X*_1_ (acetic acid), *X*_2_ (formic acid) *X*_3_ levulinic acid), *X*_4_ (furfural), and *X*_5_ (vanillin), while inhibitory intensities were inserted at *X*-axis. However, tolerance of yeast strain to inhibitors varies with experimental conditions, such as temperature, pH, and components of the medium. Experiments were performed at a confidence interval of around 90–95%.

### Observed and predicted values of biomass and ethanol randomly obtained by applying the fractional factorial design 2^5−2^

Fractional factorial planning has been used as screening factor in order to eliminate factors that show little effect on the responses. In Table 2, effects of aleatory combinations were determined considering the following five inhibitors: *X*_1_ (acetic acid), *X*_2_ (formic acid), *X*_3_ (levulinic acid), *X*_4_ (furfural), and *X*_5_ (vanillin). Such inhibitors were selected to compare values of biomass and ethanol production through a minimal number of experimental runs [eight runs). The highest values of ethanol yields were obtained in the second run (45.4 ± 0.4, g/L) and fifth run (70. 9 ± 3.1, g/L), respectively. The results (biomass and ethanol] shown in run two resulted from combinations between low inhibitors concentrations, except for higher formic acid concentration. On the other hand, the results shown in run five resulted from the combinations between high concentration of acetic acid (20 mmol/L) associated to low concentrations of other inhibitors. In the run seven, a null value of ethanol was obtained using high concentrations of three inhibitors (acetic acid, levulinic acid, and furfural) associated to lower concentrations of two other strong inhibitors (formic acid and vanillin). Considering the eight experimental trials, no significant differences were observed between the biomass yields in presence of inhibitors.

**Table 2:** Experimental values of ethanol and biomass resulting from the application of fractional factorial design (FFD) 2^5−2^

Runs	*X*_1_	*X*_2_	*X*_3_	*X*_4_	*X*_5_	Biomass (mg/mL)	Ethanol (g/L)
1	20	5	35	10	10	15.2 ± 0.8	18.9 ± 0.5
2	5	15	10	10	10	13.4 ± 0.1	45.4 ± 0.4
3	5	5	35	30	5	14.2 ± 0.5	34.4 ± 1.0
4	5	15	35	10	5	14.7 ± 2.2	23.2 ± 0.7
5	20	5	10	10	5	15.3 ± 2.4	70.9 ± 3.1
6	20	15	10	30	5	13.1 ± 0.7	27.7 ± 1.4
7	20	15	35	30	10	13.6 ± 1.4	0.0 ± 0.0
8	5	5	10	30	10	13.8 ± 0.1	33.7 ± 1.2

*X*_1_ (acetic acid), *X*_2_ (formic acid), *X*_3_ (levulinic acid), *X*4 (furfural), and *X*_5_ (vanillin). Data are mean ± SD resulted from experimental repetitions

### Effects of the three major inhibitors added to the SD-medium on the yields of ethanol and biomass by applying the 2^3^ full-central-composite design

In Table 3, the major inhibitors added to the SD-medium were acetic acid (*X*_1_), formic acid (*X*_2_), and levulinic acid (*X*_3_) and their effects were analyzed using the 2^3^ full-central-composite design in order to obtain the observed and predicted values of ethanol (g/L) and biomass (mg/mL) in 20 different runs.

**Table 3:** Effects of the three major biomass inhibitors were acetic acid (*X*_1_), formic acid (*X*_2_), and (*X*_3_) (levulinic acid) added to the SD-medium on the yields of ethanol yields and biomass by applying the 2^3^ full-central-composite design

Runs	*X*_1_	*X*_2_	*X*_3_	Biomass (mg/mL)		Ethanol (g/L)	
				Observed values	Predicted values	Observed values	Predicted values
1	5.0	25.0	10.0	12.4 ± 0.6	12.4	61.1 ± 1.5	60.7
2	10.0	25.0	10.0	11.6 ± 0.6	11.6	49.8 ± 4.9	51.9
3	5.0	35.0	10.0	11.8 ± 0.1	11.9	51.2 ± 2.2	49.4
4	10.0	35.0	10.0	11.2 ± 0.3	11.2	33.1 ± 3.6	37.2
5	5.0	25.0	20.0	12.2 ± 0.5	12.2	44.3 ± 2.5	41.4
6	10.0	25.0	20.0	11.2 ± 0.8	11.1	31.9 ± 2.8	34.7
7	5.0	35.0	20.0	11.3 ± 0.1	11.3	41.9 ± 5.3	40.8
8	10.0	35.0	20.0	10.3 ± 0.1	10.6	29.1 ± 2.6	30.7
9	3.3*	30.0	15.0	12.7 ± 0.7	12.6	46.7 ± 2.5	50.9
10	11.7*	30.0	15.0	11.1 ± 0.5	11.1	40.9 ± 4.1	35.1
11	7.5	21.6*	15.0	11.9 ± 0.5	11.9	52.1 ± 1.7	51.7
12	7.5	38.4*	15.0	11.0 ± 0.4	10.9	39.9 ± 2.3	38.8
13	7.5	30.0	6.6*	11.7 ± 0.8	11.7	52.8 ± 3.3	50.9
14	7.5	30.0	23.4*	10.8 ± 0.2	10.8	28.9 ± 1.9	29.2
15	7.5	30.0	15.0	10.7 ± 0.2	11.0	37.0 ± 2.2	33.2
16	7.5	30.0	15.0	11.2 ± 0.5	11.0	34.2 ± 1.4	33.2
17	7.5	30.0	15.0	11.2 ± 0.2	11.0	32.7 ± 1.0	33.2
18	7.5	30.0	15.0	10.6 ± 0.3	11.0	32.4 ± 1.1	33.2
19	7.5	30.0	15.0	11.5 ± 0.7	11.0	29.2 ± 0.4	33.2
20	7.5	30.0	15.0	11.0 ± 0.1	11.0	33.1 ± 0.1	33.2

Fermentations were performed using strain the yeast strain 63 M and the inhibitors (mmol/L) were as follows: *X*_1_ (acetic acid), *X*_2_ (formic acid), and *X*_3_ (levulinic acid), while the stars (*) indicates Axial points

In the first run the observed value of ethanol (61.1 ± 1.5 g/L) was close to its predicted value (60.7 g/L), whereas small differences were also observed in other three runs (fifth, seventh, tenth, and seventeenth). In the other runs, predicted values were lower than the observed values. Also on the same Table 3), the six axial tests indicated by stars (*, distance from the center point) allowed to compare and analyze the effects of the concentration of three main inhibitor on the predicted values of ethanol in the presence of three pairs of inhibitors of low and high concentrations.

### Model of ethanol production obtained with variance analysis (ANOVA)

ANOVA test is used to find differences between means of three or more groups of experiments; However, ANOVA simply indicates that there are differences between means, but does not indicate which was more significant.

In Table 4, analysis of variance (ANOVA) provides values that indicate the better adjustment of the mathematical model obtained for ethanol production. In addition, *P*-value indicated the incompatibility of the means in relation to the statistical model. The larger the magnitude of the *F*-value, the smaller is the -value. Nevertheless, the *P*-value does not seem to be well-understood [22], while the fitting of the experimental data to the regression model indicates that the variations in the ethanol concentration were also not well-explained by the model equation [23].

**Table 4:** Analysis of variance (ANOVA) for the quadratic model analysis for ethanol production

	Degree of freedom (*DF*)	Sequential sum of squares	Adjusted means of square	*F*-value	*P*-values (probability)
Regression	9	1530.06	170.006	11.35	0.001
Linear	3	1072.07	122.088	8. 15	0.006
Square	3	392.06	130.686	8.72	0.005^b^
Interaction	3	65.93	21,977	1.47	0.288
Residual error	9	134.83	14.981	–	–
Lack- of- fit	5	101,89	20.378	2.47	0.200
Pure error	4	32.94	8.234	–	–
Total	18	–	–	–	–

SD-medium containing initially 2% yeast extract, 18% glucose and 10 mg/mL of starting cells during fermentation periods of 6 h at 34 °C

Also in Table 4, the significant terms of the mathematical model equation obtained for the ethanol production gave *P* > 0.050, while the lack of adequacy of the ethanol production model was not significant. However, results described indicate that the independent variables seem to explain the changes in ethanol production by the yeast cells. Thus, the quadratic regression model seems to better represent the ethanol production. To describe the influences of the factors (inhibitors) on ethanol (Eq. 1) and biomass yields (Eq. 2), the 2^3^ full factorial design with center points was applied. The Reponses *Y*_1_ (ethanol) and *Y*_2_ (biomass) were expressed as follows:

1 $$\left( {\text{a}} \right)Y_{ 1} \left( {\text{ethanol}} \right) = 3 3 3. 6- 1 2. 1X_{ 2} - 7. 8X_{ 3} + 0. 6 { }X_{ 1}^{ 2} + 0. 2X_{ 2}^{ 2} + 0. 1X_{ 3}^{ 2}$$

where *Y*_1_ is the predicted response for the production of ethanol obtained in the presence of formic acid (*X*_1_), levulinic acid (*X*_2_), and furfural (*X*_3_), respectively.

2 $$\left( {\text{b}} \right)Y_{ 2} \left( {\text{biomass}} \right) = 20. 9 2- 0. 8 5X_{ 1} - 0.0 5X_{ 1}^{ 2}$$

where *Y*_2_ is the predicted response (biomass production) obtained in the presence of formic acid (*X*_1_). The model equations were obtained at a confidence level of 95% (Tukey test).

### RSM analysis

**Fig. 3:**
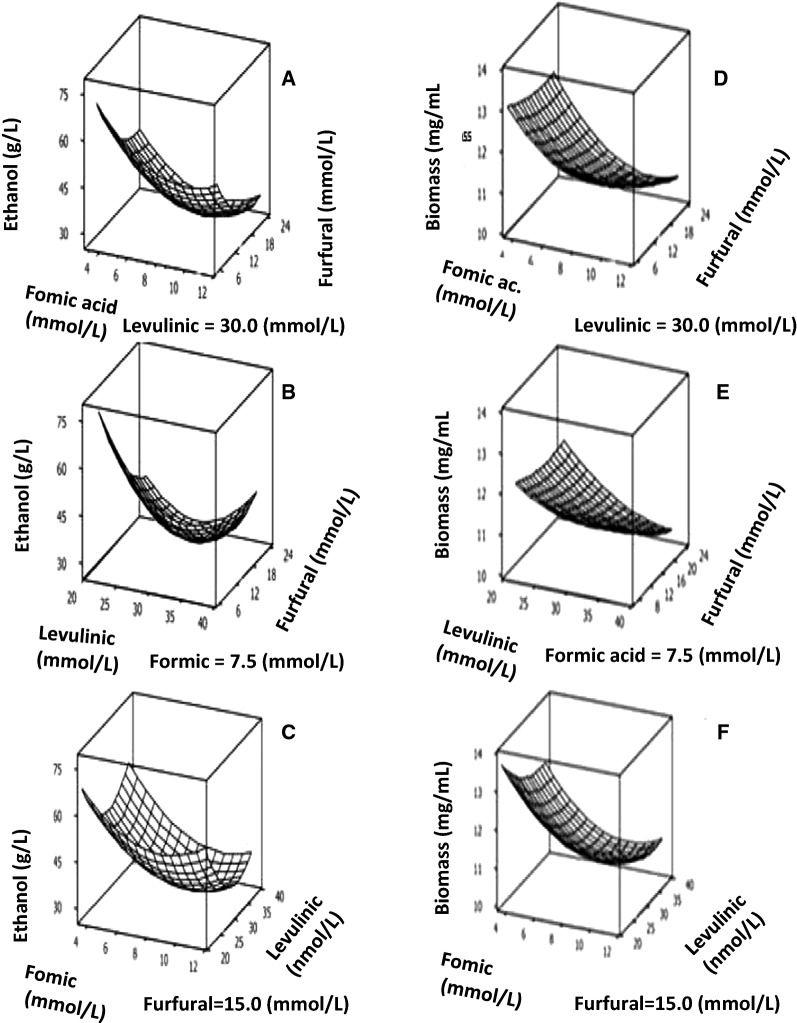
RSM analyses showing the positive interactions originated from associations between decreasing concentrations of two inhibitors added to the SD-medium on ethanol formation (Parts A to C) and growth (Parts D to F)

In Fig. 3, increases in the positive interactions between two different inhibitors on ethanol (Parts A to C) and biomass formation (Parts D to F) were observed in presence of a fixed concentration of a third inhibitor.

Concerning ethanol production, positive interactions between inhibitors were as follows (Fig. 3):

- Part A—between formic acid (varying from 4 to 12 mmol/L) and furfural (varying from 6 to 24 mmol/L) with levulinic acid fixed at 30 mmol/L;
- Part B—levulinic acid (varying from 20 to 40 mmol/L) and furfural (varying from 6 to 24 mmol/L) with formic acid fixed at 7.5 mmol/L;
- Part C—formic acid (varying from 4 to 12 mmol/L) and levulinic acid (varying from 20 to 40 mmol/L) with furfural fixed at 15 mmol/L;

In respect to biomass formation the effects of positive interactions between inhibitors were as follows:

- Part D—formic acid (varying from 4 to 12 mmol/L) and furfural (varying from 6 to 24 mmol/L) with levulinic acid fixed at 30 mmol/L;
- Part E—levulinic acid (varying from 20 to 40 mmol/L) and furfural (varying from 8 to 24 mmol/L) with formic acid fixed at 7.5 mmol/L;
- Part F—formic acid (varying from 4 to 12 mmol/L) and levulinic acid (varying from 12 to 40 mmol/L) with furfural fixed at 15 mmol/L;

Thus, increases in positive interactions between inhibitors were proportional to reductions in values of inhibitor concentrations.

### Interactions between low concentrations of inhibitors based on the response optimizer

As shown in Table 5, increases in production of biomass and ethanol were obtained with the addition of low concentrations of inhibitors to the SD-medium. The maximal predicted responses were 70.1 ± 1 g/L for ethanol and 13.1 ± 1.0 mg/mL for biomass, while obtained responses were 72.6 ± 1 g/L for ethanol and 13.2 ± 0.1 mg/mL for biomass. Global desirability (value of 1.000) is extensively used to simultaneously optimize multiple responses.

**Table 5:** Response Optimizer applied to optimize growth and ethanol responses at low concentrations of inhibitors added to SD-medium

Parameters	Goals	Minimum	Medium	Maximum
Ethanol (g/L)	Maximal	50	65	65
Biomass (mg/mL)	Maximal	12	13	13
Global solutions				
Formic acid = 3.3 mml/L				
Levulinic acid = 21.6 mml/L				
Furfural = 6.6 mml/L				

Predicted responses	Obtained responses			
Ethanol = 70.1 ± 1 g/L	Ethanol = 72.6 ± 1.0 g/L			
Biomass = 13.1 ± 1 mg/mL	Biomass = 13.2 ± 0.1 mg/mL			
Global desirability = 1.000				

SD-medium containing 18% glucose, 2% yeast extract, 10 mg/ml of initial yeast cell in the presence of biomass inhibitors during 6 h fermentation times

Addition of mixture of inhibitors to the SD-medium is highly important to study the resistance of yeast cells to mixtures of inhibitors usually present in biomass hydrolysates. Another relevance fact would be the possibility to assess the level of toxicity of hydrolysates. The concentrations of the inhibitors in hydrolysates calculated by the Response Optimizer were the following: 3.3 mmol/L formic acid, 21.6 mmol/L levulinic acid, and 6.6 mmol/L furfural.

**Fig. 4:**
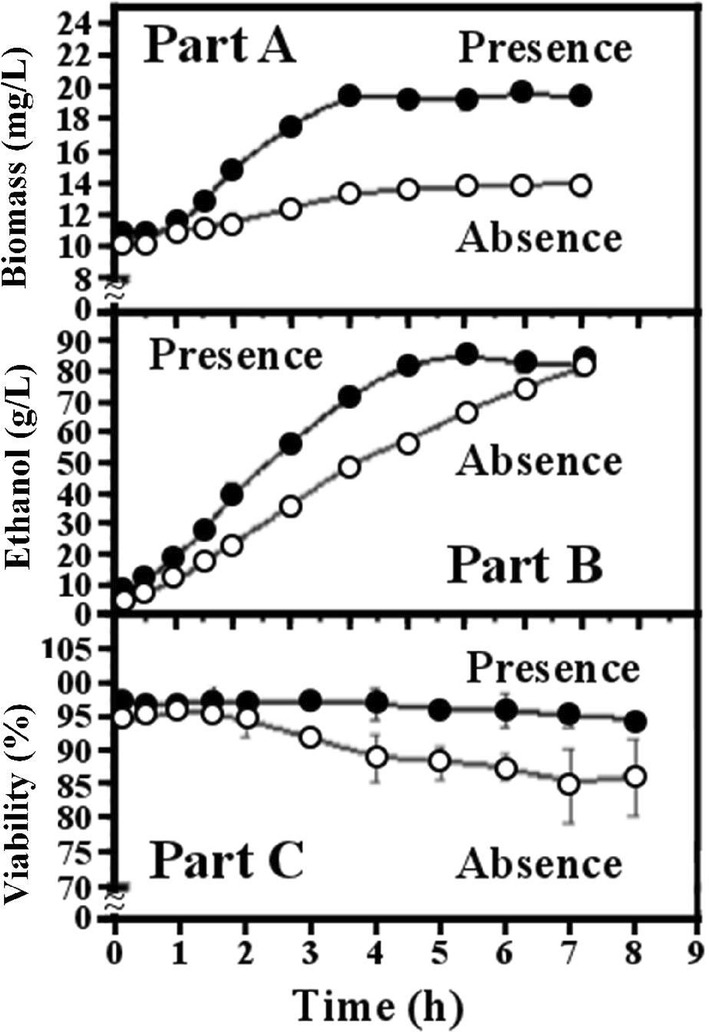
Experimental validation of fermentation responses (biomass, ethanol, viability) after 6 h in presence (Black circle) and absence (White circle) of the optimized mixture of three inhibitors (formic acid, levulinic acid, furfural)

Figure 4 describes the experimental validation of the increases in values of biomass (Part A, g/L), ethanol (Part B, g/L), and viability (Part C, %) resulted from the addition of the mixture of inhibitors (furfural, formic, and levulinic acids added together) to the SD-medium:

- In part A, initial biomass (13 g/L) increased, becoming constant (18 g/L) after 3 h fermentation, while slight increase was observed in the absence of inhibitors;
- In Part B, initial ethanol increased from 10 g/L to a constant value (80 g/L) above 5 h of fermentation, while in the absence of inhibitor, a lower rate of increase was observed between the initial value of 5 g/L up to the final concentration of 80 g/L reached in 8 h;
- In Part C, viability remained constant (95%) in presence of inhibitors, whereas in the absence of inhibitors it remained constant (95%) during 2 h and started to decrease slowly to 85% in 8 h fermentation.

However, the industrial sector makes little use of statistic methodologies to predict results and analyze data during fermentations.

## Conclusions and perspectives for future researches

Statistical methods are useful tools to predict favorable combinations between environmental conditions to improve yeast growth and fermentation. The experimental design proved to be effective in optimizing interactions between yeast responses in medium, while RSM plots or graphs allowed predicting the effects of positive interactions between low concentrations of inhibitors during growth and ethanol production. In addition, the RSM values showed increasing positive interactions between inhibitors when added at decreasing concentrations in the medium. Moreover, Response Optimizer allowed optimizing concentrations of inhibitors in a mixture of the main inhibitors (3.3 mmol/L formic acid, 21.6 mmol/L levulinic acid, and 6.6 mmol/L furfural) in order to improve the growth and ethanol production.

Improved values of growth, ethanol production resulted from statistical treatments were experimentally validated. Thus, effects of inhibitors cannot be ignored in presence of low concentrations of inhibitors, especially during the production of the 2-G ethanol.

Often times, concentration of inhibitors increase at the end in batch fermentations, and this was observed under conditions of the present work. Thus, the development of strategies, as well as the obtaining of new yeasts resistant to biomass inhibitors should be encouraged for use in the ethanol production sector. New yeast capable of expressing the gene *Haa1* are resistant to weak acids, mainly acetic acid or lactic, will be useful to the industrial sector of the ethanol production [24].

## Materials and methods

### Yeast strain

The yeast used in the present work is a strain of *Saccharomyces cerevisiae* (MAT a/α, LYS/lys, URA/ura genotype) constructed in our laboratory by hybridization between haploids derived from tetrad dissection [25].

### Media

The SD-medium was prepared to contain initially 2% yeast extract in order to enhance propagation of the yeast cells [26]. Such supplement provides essential precursors for the yeast metabolism [27]. As the optimum pH for fermentation with strains of *S. cerevisiae* is between 4.0 and 5.0 [28], the initial pH of the medium was adjusted to 4.5 with sterilized solutions of acid (HCl) and alkali (NaOH). Fermentation is performed in Brazilian alcohol factories by yeast cells at high initial concentrations of sugar (18% total reducing sugars) and high cell density (10 mg/mL yeast cells) for 8–10 h periods.

### Commercial inhibitors

Commercial inhibitors were as follows: levulinic acid from Fluka Analytical (pKa 4.66 at 25 °C); glacial acetic acid from Sigma-Aldrich (pKa 4.75 at 25 °C); vanillin from J. T. Baker; formic acid from Fluka Analytical (pKa 3.75 at 20 °C), and furfural from Sigma-Aldrich. Sterilized solutions containing inhibitors were added to the medium as required by the experiments.

### Preparation of the yeast cream

Fresh cells of stock cultures were propagated in 250 mL Erlenmeyer flasks containing 100 mL of YPD-medium (8% glucose) for propagation of the yeast cells during 16 h at 30 °C in the rotatory shaker operating at 125 rpm. After that, the cells were harvested by centrifugation (500×*g* for 6 min at 4 °C), washed in distilled water twice, then the pellet was diluted to obtain the yeast cream (around 8% dry mass, w/v). At low concentrations of sugar (8% glucose), the yeasts cells prefer to grow than to produce ethanol.

### Analytical procedures

Cell viability was determined using the methylene blue method [29], while ethanol was determined with the use of a gas chromatography (model CG-37; Instrumentos Científicos, São Paulo, Brazil). Cells were dried to a constant weight at 105 °C to obtain dry biomasses, which were expressed in g/L of medium.

### Statistical optimization of the ethanol production process

The statistical treatments used in the present work involve the 2^5−2^ fractional factorial (1/4 fractional factorial) and the 2^3^ Complete Factorial Planning (2^3^ full-central-composite design or CCD). The 2^3^ full-central-composite design was used in order to obtain the observed and predicted values of ethanol (g/L) and biomass (mg/mL). In addition, Factorial or Fractional Factorial Design (with center points) and add “star” points is commonly called a central composite design.

Fractional Factorial Planning has been used for screening to eliminate factors that show little effects on the responses. The main effects and Pareto graphs are derived from Factorial Design. Pareto graphs are used to draw conclusions about which of the variables and interactions are the most important. Pareto graphs are usually presented in the form of a column chart. The main effects indicate the ability to separate low order interactions from one another. In addition, Response Optimizer is a tool used to predict interactions between factors (inhibitors), as well as to perform optimization by increasing values of responses using a Minitab program, while Response surface methodology (RMS analysis) is one of the most important and widely applied statistical designs to develop and optimize interactions between variables. The Minitab 14 (PA, USA) software is a powerful statistical tool used to analyze data and find relevant solutions for the negative effects of inhibitors.

Table 6 describes the following independent variables selected for the present work: *X*_1_ (acetic acid), *X*_2_ (formic acid), *X*_3_ (levulinic acid), *X*_4_ (furfural), and *X*_5_ (vanillin). Real and codified values of independent variables were fixed by applying the factorial design 2^5−2^. The maximum coded values (+) varied between 10.0 mmol/L (vanillin) to 35.0 mmol/L (levulinic acid), while the minimum coded values (−) varied between 5.0 mmol/L (acetic acid, formic acid, and vanillin) to 10 mmol/L (levulinic acid).

**Table 6:** Estimated coefficients for real and codified values of independent variables (inhibitors) were fixed by applying the fractionated factorial design 2^5−2^

Variables (mmol/L)	Symbols	Values and ranges (mmol/L)	
		(− 1)	(+ 1)
Acetic acid	*X*_1_	5.0	20.0
Formic acid	*X*_2_	5.0	15.0
Levulinic acid	*X*_3_	10.0	35.0
Furfural	*X*_4_	10.0	30.0
Vanillin	*X*_5_	5.0	10.0

*X*_1_ (acetic acid), *X*_2_ (formic acid), *X*_3_ (levulinic acid), *X*_4_ (furfural), and *X*_5_ (vanillin)

## Data Availability

The datasets used and/or analyzed during the current study are available from the corresponding author on reasonable request.

## Abbreviations

2-G: second-generation
RSM: response surface methodology
CCD: central composite design
YPD: yeast extract–peptone–dextrose
SD: synthetic defined: medium containing glucose, yeast nitrogen-base (without amino phenylalanine, uracil and adenine)
ANOVA: analysis of variance
HMF: hydroxy-methylfurfural
